# Intrinsic factor recognition promotes T helper 17/T helper 1 autoimmune gastric inflammation in patients with pernicious anemia

**DOI:** 10.18632/oncotarget.26874

**Published:** 2019-04-23

**Authors:** Arianna Troilo, Alessia Grassi, Luisa Petrone, Fabio Cianchi, Marisa Benagiano, Chiara Della Bella, Nagaja Capitani, Jacopo Bitetti, Sofia D’Elios, Simona Tapinassi, Annalisa Azzurri, Heba Alnwaisri, Jacopo Romagnoli, Nicola Bizzaro, Mathijs Bergman, Cosima Tatiana Baldari, Mario Milco D’Elios

**Affiliations:** ^1^ Department of Experimental and Clinical Medicine, University of Florence, Florence, Italy; ^2^ Endocrinology Unit, Careggi Hospital, Florence, Italy; ^3^ Department of Surgery, University of Florence, Florence, Italy; ^4^ Department of Life Sciences, University of Siena, Siena, Italy; ^5^ Department of Clinical and Experimental Medicine, University of Pisa, Pisa, Italy; ^6^ Toscana Centro Hospital, Firenze-Prato, Italy; ^7^ Surgery Unit, Rome Catholic University, Italy; ^8^ San Antonio Hospital, Tolmezzo, Italy; ^9^ Amsterdam Institute for Molecules, Medicines and Systems, Vrije University, Amsterdam, The Netherlands

**Keywords:** intrinsic factor, interferon-gamma, interleukin-17, pernicious anemia, atrophic gastritis

## Abstract

The intrinsic factor is the major humoral autoantigen in pernicious anemia/autoimmune gastritis. Although many studies have examined the autoantibody response to intrinsic factor and H^+^,K^+^-ATPase, no information is available on possible pathogenic mechanisms mediated by intrinsic factor - specific gastric T cells. Aim of this study was to investigate intrinsic factor-specific T cells in the gastric mucosa of pernicious anemia patients and define their functional properties. For the first time we provide evidence that gastric mucosa of pernicious anemia patients harbour a high proportion (20%) of autoreactive activated CD4^+^ T-cell clones that specifically recognize intrinsic factor. Most of these clones (94%) showed a T helper 17 or T helper 1 profile. All intrinsic factor-specific clones produced tumor necrosis factor-α, interleukin-21 and provided substantial help for B-cell immunoglobulin production. Most mucosa-derived intrinsic factor-specific T-cell clones expressed cytotoxicity against target cells. Our results indicate that activation of intrinsic factor-specific T helper 17 and T helper 1 T cells in the gastric mucosa represent a key effector mechanism in pernicious anemia suggesting that the T helper 17/T helper 1 pathway may represent a novel target for the prevention and treatment of the disease.

## INTRODUCTION

Pernicious anemia (PA) is an haematological disorder characterized by immunological and gastric pathologies [1, 2]. PA is an autoimmune disease consisting of gastric atrophy, macrocytic anemia, and reduction of parietal cells producing the intrinsic factor which is essential for vitamin B12 absorption. Vitamin B12 is required for erythropoietic process and synthesis of myelin [3]. The autoreactive process of PA proceeds during decades up to a severe state associated with a lack of vitamin B12 starting from a mild chronic autoimmune body gastritis (AIG). PA is usually characterized by anemia of the megaloblastic type and gastric atrophy which can be anticipated by neuropathies. The presence in PA of autoantibodies against intrinsic factor (secreted by the gastric parietal cells), and the coexistence in PA patients of other autoimmune disorders (e.g. autoimmune thyroiditis) support the immunopathological autoimmune bases of PA and AIG [4]. PA and AIG patients have documented both gastric mucosal infiltration of activated CD4+ T cells, macrophages and atrophy of the gastric corpus [5].

Intrinsic factor (IF) and gastric H+,K+-adenosine triphosphatase (ATPase) are crucial autoantigens recognized by autoreactive B cells and autoantibodies in PA patients [2, 6]. In experimental autoimmune gastritis (EAIG) the gastric H+,K+-ATPase is also the major autoantigen needed to elicit an organ-specific autoimmune disease [7–9]. In all the models of AIG H+,K+-ATPase stimulates both the production of autoantibodies and gastric inflammation, which is usually followed by loss of acid-secreting parietal cells and zymogenic cells [10, 11]. The immunohistopathologic lesions of EAIG (consisting of T and B cells, and macrophages) are very similar to those observed in patients with pernicious anemia [12]. No study have up to now unraveled the type of gastric T-cell responses specific for IF in PA.

We have characterized indeed in this research project the effector functions of T cells specific for intrinsic factor. Overall, we demonstrate that in PA patients, gastric-derived T helper cells were able to secrete interferon gamma and interleukin 17 in response to IF and suggest that IF drives a local Th17/Th1 inflammatory response, which may represent a novel target for PA treatment.

## RESULTS

### Gastric mucosa of PA patients harbour autoreactive T helper cells specific for IF

We studied activated T cells derived from the stomach of patients with PA. We recovered 265 T helper clones and 34 T suppressor clones from the gastric mucosae of 7 women affected with PA. We investigated at clonal level the proliferative responses in response to medium or IF, of all gastric PA-derived CD4^+^ and CD8^+^ T cells. No CD8^+^ T cells clone proliferated in response to IF although they showed proliferation to PHA.

**Figure 1:**
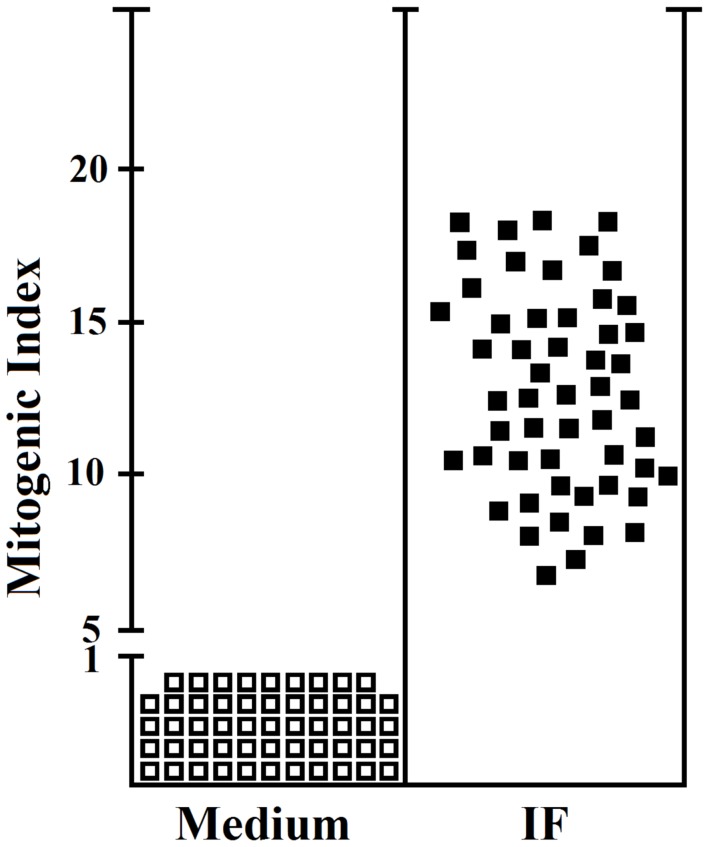
Proliferative response to IF of the IF-specific gastric CD4^+^ T cell clones obtained from PA patients. All the 265 CD4^+^ and 34 CD8^+^ T cell clones obtained from the gastric mucosae of the 7 PA patients were assayed for proliferation in response to medium (o), or IF (10 nM) (■), in the presence of irradiated autologous presenting cells, by measuring [^3^H]thymidine uptake after 60 h of coculture. 53 CD4^+^ T-cell clones significantly proliferated to IF and their mitogenic index are shown in the figure. All the experiments have been performed in triplicates.

20% (53) of gastric-derived CD4^+^ T cells showed proliferation to IF (Figure 1). Each PA patient displayed a comparable percentage of T helper cells proliferating to IF. On the other hand, we obtained 237 T helper and 32 T suppressor clones from the stomach mucosae of *Helicobacter*-infected patients, seronegative for anti-intrinsic factor antibody. We evaluated in all patients the proliferative response to IF of both gastric CD4^+^ and CD8^+^ T cells in MHC-restricted conditions. While CD4^+^ and CD8^+^ T cell clones were activated by PHA, none proliferated to IF. We have also investigated in PA patients the amount of T cells specific for IF present in the peripheral blood (pb) and compared it with the one found in the gastric mucosa. In the pb of PA patients the frequency of IF-specific T cells found in the peripheral blood ranged between 1:1800 and 1:3300 while in gastric mucosa it was remarkably higher (nearly 25%).

**Figure 2:**
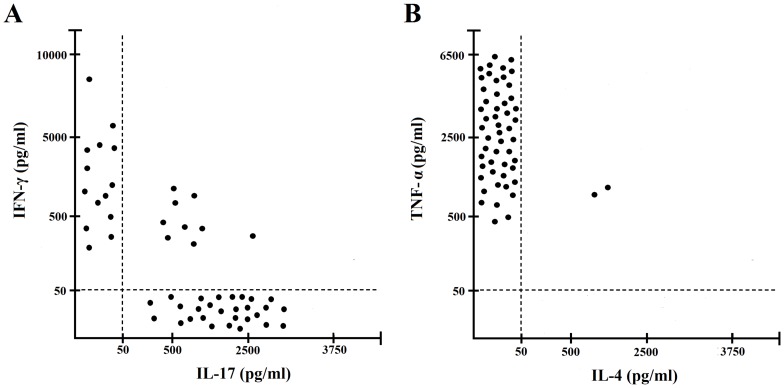
Cytokine profile of gastric IF-specific CD4+ T cell clones obtained from PA patients. Th clones were tested for cytokine production (**A**, **B**). IF-specific Th clones were stimulated with IF and IFN-γ and IL-17 TNF-α and IL-4, production was measured in culture supernatants. In unstimulated cultures, levels of IFN-γ and IL-17 TNF-α, IL-4, were consistently < 20 pg/ml. All the experiments have been performed in triplicates.

We also investigated the cytokine profile of gastric T cells specific for IF, using IF and autologous APCs and cytokine production was measured in supernatants. Upon antigen stimulation with IF, 28 IF-specific T-cell clones were polarized Th17 clones, 14 Th clones were polarized Th1, 9 Th clones were Th17/Th1 and only 2 were Th0 clones (e.g. secreting both IL-4 and IFN-γ) (Figure 2). TNF-α was produced by nearly all T cells specific for IF.

**Figure 3:**
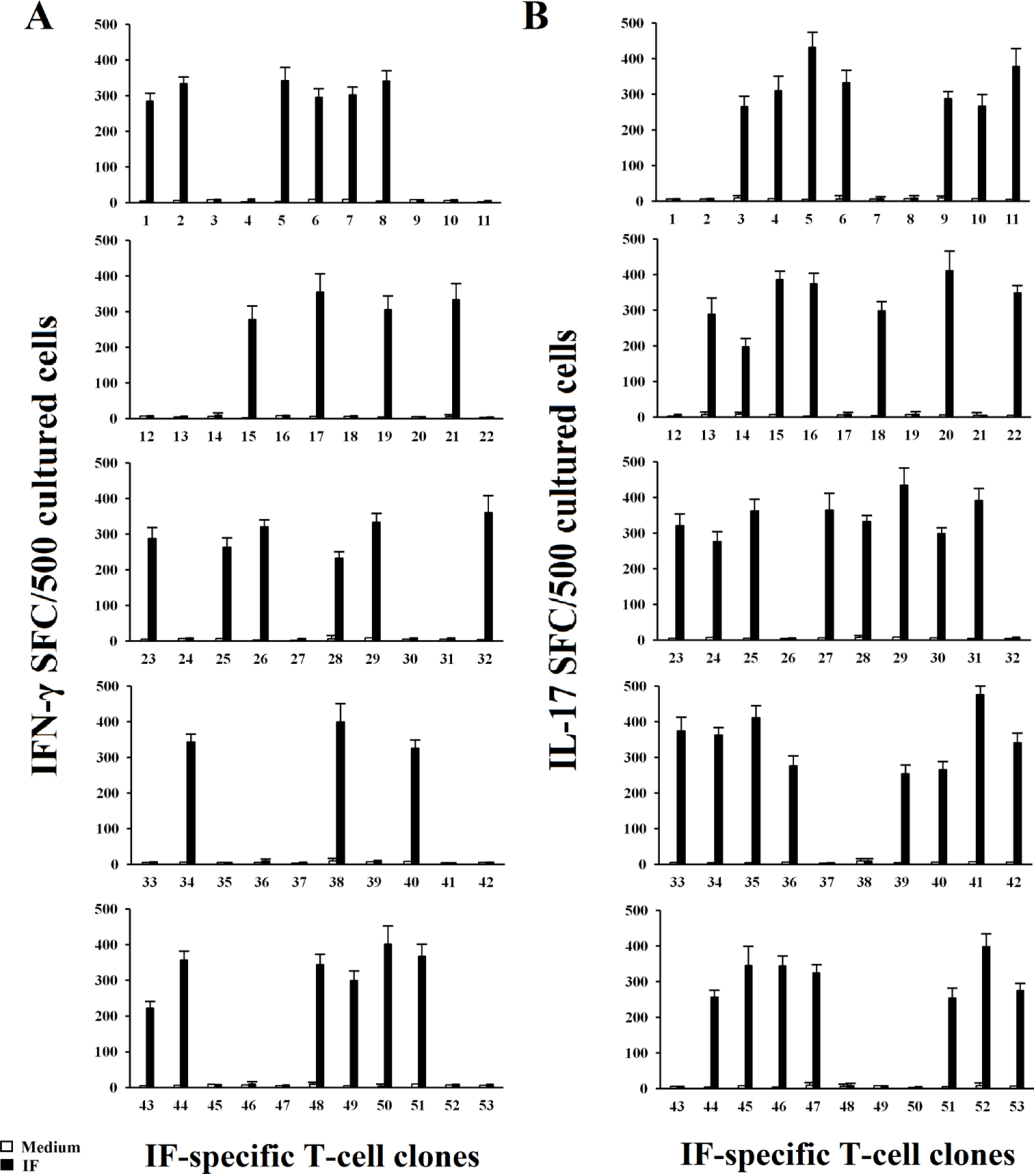
IF driven IFN-γ and IL-17 secretion by IF-specific gastric derived Th clones from PA patients. Numbers of IFN-γ spot-forming cells (SFCs) after stimulation of gastric T cell clones with medium alone, or IF (**A**). T cell blasts from each clone were stimulated for 48 h with medium alone (o), or IF (■), in the presence of irradiated autologous APCs in ELISPOT microplates coated with anti-IFN-γ antibody. IFN-γ SFCs were then counted by using an automated reader. After specific stimulation, 25/53 IF-specific gastric T cell clones produced IFN-γ. Values are the mean ± SD number of SFCs per 10^5^ cultured cells over background levels. All the experiments have been performed in triplicates. Numbers of IL-17 spot-forming cells SFCs after stimulation of gastric T cell clones with medium alone, or IF (**B**). T cell blasts from each clone were stimulated for 48 h with medium alone (o), or IF (■) in the presence of irradiated autologous APCs in ELISPOT microplates coated with anti-IL-17 antibody. IL-17 SFCs were then counted by using an automated reader. After specific stimulation 37/53 IF-specific gastric T cell clones produced IL-17. Values are the mean ± SD number of SFCs per 105 cultured cells over background levels.

All 53 T helper clones specific for IF obtained from gastric mucosae of patients with PA were further screened by IFN-γ and IL-17 ELISPOT in response to IF. Upon appropriate antigen stimulation using ELISPOT specific and very sensitive technique, it is of note that thirty-seven gastric-derived CD4^+^ T cell clones produced IL-17, and twenty-five clones produced IFN-γ (Figure 3). Interestingly, all IL-17-producing IF-reactive T cell clones produce IL-21 (mean ± SE per 10^6^ T cells was 3.4 ng/ml ± 0.6) in response to antigen stimulation.

### Helper function for antibody production by gastric-derived T cells specific for IF

We evaluated the ability of T cells specific for IF to help antibody synthesis of B cells in the presence of IF in a 10 day T-B helper assay for antibody production.

**Figure 4:**
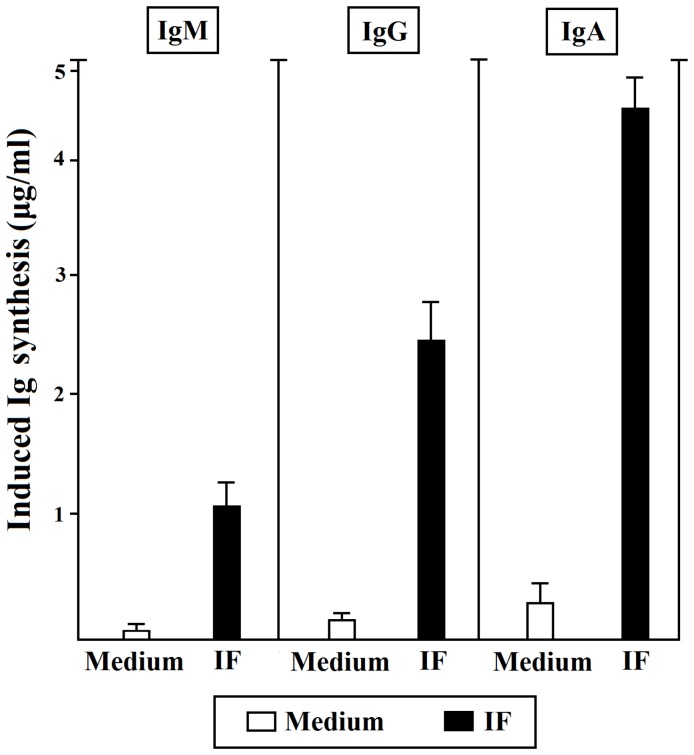
Helper function of gastric IF-specific T cells derived from PA patients. Gastric IF-specific T cell clones help Ig production by autologous B cells. Autologous peripheral blood B cells (5 × 10^4^) were co-cultured with IF-specific T cell blasts (5 × 10^4^) in the absence (o) or presence of IF (■). After 10 days, cell free culture supernatants were harvested and tested for the presence of IgM, IgG, and IgA by ELISA. The results represent the mean (±SE) Ig levels induced by T cell clones over the spontaneous Ig production in cultures of B cell alone. All the experiments were done in triplicates.

All the T cells specific for IF were able to help the antibody production by autologous B cells in the presence of IF (Figure 4).

### Gastric-derived IF-specific T cells display cytotoxicity against gastric mucosal cells and B cells

**Figure 5:**
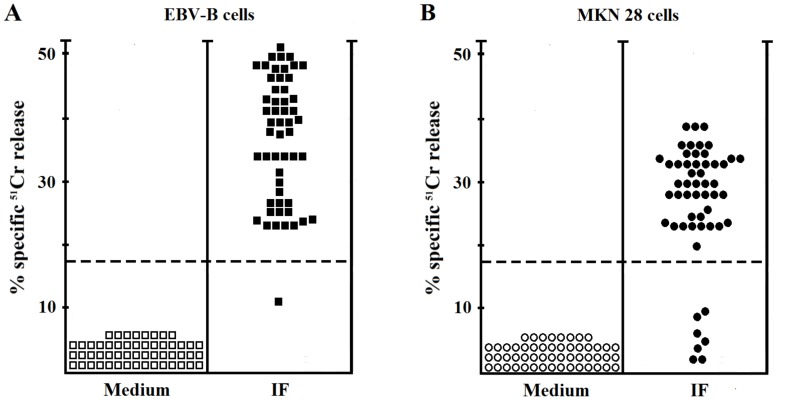
Cytotoxic activity of IF-specific gastric-derived T cells derived from PA patients. (**A**) To assess their cytotoxicity, IF-specific T cell clones were co-cultured at an effector-to-target ratio of 10 to 1 with ^51^Cr-labeled autologous EBV-B cells pulsed with IF (●) or medium (○) alone. ^51^Cr release was measured as index of specific target cell lysis. (**B**) To assess their ability to induce killing of gastric target cells, IF-specific T cell clones stimulated with IF (●) or medium (○) alone were co-cultured with ^51^Cr-labeled gastric MKN 28 cells at an effector-to-target ratio of 10 to 1, and ^51^Cr release was measured as index of c target cell death.

We evaluated the cytotoxicity of gastric-derived IF-autoreactive T cells by testing the ability of each T-cell clone to kill either autologous B cells or gastric mucosal MKN 28 cells. The perforin-related cytotoxicity was belonging to Th17, Th1 Th17/Th1, and Th0 T-cell clones (Figure 5A).

To assess if gastric T cells might exert killing of gastric epithelial cells, gastric mucosal cells such as MKN 28 pulsed with intrinsic factor were used following co-cultured with ^51^Cr-MKN 28 and T cells (at an Effector/Target ratio of 10 / 1 for 18 h in the presence of IF) (Figure 5B). Upon IF activation, the 14 Th1, 23/28 Th17, 8/9 Th17/Th1, and 1 out of 2 Th0 clones were able to induce death.

## DISCUSSION

The main findings of this work are that in the gastric mucosa of patients with pernicious anemia intrinsic factor: a) is able to drive T helper 17 and T helper 1 T-cell responses; b) is capable to promote T-cell help for antibody production; c) activates T-cell cytotoxicity against gastric epithelial cells. The experimental approach used in this study has been successfully used in many other immunopathological conditions [13, 15–17]. Each PA patient, but not *H. pylori*-infected patient seronegative for IF, displayed in the gastric mucosa a significant proportion of gastric T cells specific for IF. Furthermore all PA patients, that were not infected by *H. pylori*, had no T cells specific for *H. pylori* in their gastric mucosae.

It is well known that IF is able to activate autoreactive B cells and their anti-IF autoantibody production. This study highlights that IF is not only able to promote IF-specific autoantibodies but also gastric T-cell inflammation. Gastric-derived T cells of PA patients were able to secrete many cytokines such as IL-17, IL-21, TNF-α and IFN-γ, but not interleukin 5 nor interleukin 4. Using the same clonal experimental approach, a clear-cut Th2 cytokine profile of T cells was found in parasitic infestations, such as in patients with *Schistosoma mansoni* infection [16, 18].

Furthermore we found that IF was able also to activate the helper function to B cells by T cells specific for IF, suggesting that the anti-IF autoantibody production might be at least in part favored by gastric mucosal T cell help to autologous B cells [19–23].

We also demonstrated that gastric-derived T cells specific for IF were able to kill both gastric mucosal cells and B cells, indicating that T-cell cytotoxicity is another important mechanism potentially leading to death of gastric epithelial cells and gastric atrophy. It is possible indeed to speculate that B7.1 and B7.2 might be up-regulated by IFN-γ in gastric epithelial cells of PA patients and that those co-stimulatory molecules together with IL-17, IL-21 and TNF-α might favor the continuous chronic activation of mucosal T cells in the stomach of PA patients [24]. It is of note that in PA patients there are high serum levels of TNF-α compared to healthy subjects [25].

Given that most of the IF-specific T cells found at gastric level secreted interleukin 21 and interleukin 17 we can speculate that Th17 cytokines are very important for driving gastric autoimmunity and autoantibody production in PA patients, as it is the case in many autoimmune diseases [21, 26–33]. We can hypothesize that different putative mechanisms of gastric damage might be activated by intrinsic factor-activated Th1 and Th17 T cells. Reports that gastric resident dendritic cells and different antigens, such as *H. pylori,* were able to activate T cells [34–38] suggest that resident gastric dendritic cells may also be implicated in the activation of intrinsic factor reactive T cells following activation by IF, as in other autoimmune pathologies [27, 30, 39–42].

In conclusion, our data provide evidence and indicate that activation of IF-specific Th17 and Th1 effectors represent key components of disease in PA suggesting that the Th17/Th1 pathway may represent a novel therapeutic target for PA.

## MATERIALS AND METHODS

### Reagents

Human intrinsic factor was purchased by Prospec (Ness Ziona, Israel). Human recombinant (hr) interleukin (IL)-2 were provided by Novartis, Siena, Italy. PHA was purchased from Life Technologies (Carlsbad, CA). Fluorochrome-conjugated human monoclonal antibodies anti-CD3, anti-CD4, anti-CD8, anti-IFN-γ, anti-TNF-α, and isotype-matched control mAb were purchased from BD Biosciences (San Jose, CA, USA). The fluorochrome-conjugated anti-IL-17 mAb was obtained from eBioscience (San Diego, CA, USA). PMA, ionomycin and brefeldin A were purchased from BD Biosciences (San Jose, CA, USA).

### Patients

Upon approval of the local Ethical Committee, seven patients (5 females and 2 males, mean age 52; range 37–64 years) with PA and type A chronic AIG and 7 patients (5 females and 2 males, mean age, 51; range, 38–63 years) with *Helicobacter pylori*–induced uncomplicated type B chronic gastritis without atrophy (Hp-CG) were included in the present study. Diagnosis was made based on medical history, macrocytic anemia in peripheral blood, erythroblastosis with megaloblastic changes in bone marrow, low serum levels of vitamin B 12 and clinical responsiveness to vitamin B 12 therapy. All PA patients had serum intrinsic factor autoantibodies, as assessed by EliA (Thermo Fisher). All PA patients had negative results on both serology and 13C-urea breath test, which assess previous and/or actual *H. pylori* infection. The 7 control patients with Hp-CG were infected with CagA+VacA+*H. pylori* type I strains and were positive for anti-CagA serum immunoglobulin (Ig) G antibodies but they were negative for IF or other organ-specific autoantibodies. None of the patients had infections or inflammation. Following informed consent, biopsy specimens were obtained from the gastric mucosae and were used for diagnosis and culture of mucosa-infiltrating T lymphocytes.

### Generation and characterization of gastric T cell clones

Biopsy specimens were cultured for 7 days in RPMI 1640 medium supplemented with IL-2 (50 U/mL) to expand *in vivo*–activated T cells. Mucosal specimens were then disrupted, and single T-cell blasts were cloned under limiting dilution (0.3 cells/well), as described previously. To assess their phenotype profile, T cell clones were screened by flow cytometry with fluorochrome-conjugated anti-CD3, anti-CD4, anti-CD8 on a BD FACSCanto II (BD Bioscience), using the FACS Diva 6.1.3.software. Each clone was screened for responsiveness to IF (10 μg/ml) by measuring [^3^H]thymidine uptake after 60 hours of stimulation with medium, or IF in the presence of irradiated autologous mononuclear cells as antigen-presenting cells (APCs). The mitogenic index (MI) was calculated as the ratio between mean values of counts per minute (cpm) obtained in stimulated cultures and those obtained in the presence of medium alone. MI >5 was considered as positive.

### Assessment of T cell clones cytokine profile

To assess the cytokine production of IF-specific T cell clones upon antigen stimulation, 5 × 10^5^ T cell blasts of each clone were co-cultured for 48 h in 0.5 ml of serum-free medium with 5 × 10^5^ irradiated autologous PBMCs in the absence or presence of IF [13]. At the end of the culture period, duplicate samples of each supernatant were assayed for their IFN-γ, TNF-α, IL-4, IL-21 and IL-17 (BioSource International, Camarillo, CA) production by ELISA. For further investigation, T cell blasts from each IF-specific T cell clone were stimulated with medium or IF (10 μg/ml) in the presence of autologous APCs for 48 h in ELISPOT microplates coated with anti- IFN-γ or anti-IL-17 antibody, respectively (eBioscience, Inc., San Diego, Ca, USA). At the end of culture period, the number of IFN-γ and IL-17 SFCs were counted as described [21].

### Evaluation of antigen-specific T cell-help for functional activation of autologous B cells

The cell culture system used for the induction of Ig synthesis was performed in duplicates tubes by using complete medium supplemented with 10% FCS, as described [14]. B cells (5 × 10^4^) were cultured alone or with autologous clonal T cell blasts (5 × 10^4^) in the absence or presence of IF antigen. After 10 days, culture supernatants were collected and assayed for their Ig content, as previously described [14].

### T cell clone-mediated cytotoxicity

T cell clones cytolytic activity was assessed as reported [15]. T cell blasts of IF-specific T cell clones were incubated at ratios of 10 to 1 with ^51^Cr-labeled autologous Epstein-Barr virus transformed (EBV)-B cells pre-incubated with IF (10 mg/ml) or medium alone or human albumin (5 μg/mL). After centrifugation, microplates were incubated for 8 h at 37° C, and 0.1 ml of supernatant was removed for the measurement of ^51^Cr release, as reported [15]. The ability of IF-specific T cell clones to induce death of gastric cells was assessed using gastric mucosal MKN 28 cells as target, after activation of T cells with IF (10 mg/ml)and autologous antigen presenting cells. T cell blasts from each clone were co-cultured with ^51^Cr-labeled MKN 28 cells at an effector/target (E:T) ratio of 10 to 1 for 18 h.

### Statistical analysis

Student’s *t*-test was used for statistical analysis of the differences between experimental groups. *P* values less than or equal to 0.05 were considered significant.
